# Patient brokering in for-profit substance use disorder treatment: a qualitative study with people with opioid use disorder and professionals in the field

**DOI:** 10.1186/s12913-023-10217-z

**Published:** 2023-11-06

**Authors:** Sarah E. Clingan, Brittany M. D’Ambrosio, Peter J. Davidson

**Affiliations:** 1 Department of Psychiatry and Biobehavioral Sciences, University of California, Los Angeles, CA 90024 USA; 2 Department of Medicine, University of California, 9500 Gilman Drive, San Diego, La Jolla, CA 92093 USA; 3 School of Social Work, College of Health and Human Services, San Diego State University, 5500 Campanile Drive, San Diego, CA 92182 USA

**Keywords:** Opioid Overdose, Substance use treatment, Patient brokering, Barriers to treatment, Opioid use disorder

## Abstract

**Background:**

Opioid use and opioid overdose deaths are at an all-time high and evidence-based treatments for people with opioid use disorder (OUD) are underutilized. Therefore, we sought to understand experiences and perceptions of abuses in the for-profit substance use disorder treatment industry that could potentially put people with OUD at an increased risk for an overdose.

**Methods:**

One-on-one semi-structured interviews were conducted from November 2018 to May 2019 in Southern California with 20 people with OUD and 20 professionals who work in the substance use disorder (SUD) treatment field. A grounded theory approach was conducted to discover emerging patterns from the data.

**Results:**

Three major themes emerged:1) financial and material enticements, 2) encouraging substance use in the for-profit treatment sector, and 3) contributors to overdose risk. Participants reported that patient brokers would pay for plane tickets and offer financial incentives (e.g., money) to attract individuals to SUD treatment, capitalizing on insurance profits despite initial expenses. Participants reported being encouraged to use drugs before treatment to meet insurance conditions, thus jeopardizing genuine recovery efforts and adding to the temptation of drug use. Many participants linked patient brokering to increased overdose deaths, emphasizing the dangerous practices of brokers providing drugs, promoting relapse, and creating a revolving door of treatment, which compounds the overdose risk after periods of abstinence.

**Conclusions:**

Patient brokering and unethical abuses in the for-profit treatment industry have caused some people with OUD to seek treatment for money and housing instead of seeking treatment to stop opioid use. The harmful treatment environment was seen as a barrier to care and an unwanted obstacle to overcome on the path to recovery.

**Supplementary Information:**

The online version contains supplementary material available at 10.1186/s12913-023-10217-z.

## Background

While opioid consumption and related deaths reach record highs, evidence-based treatments for those with opioid use disorder (OUD) remain inadequately used [1, 2]. According to the latest statistics, among individuals who are 12 years of age or older, 2.7 million people in the United States had OUD in the past year [3]. Provisional data from the CDC estimated 105,752 people died from a drug overdose in the 12-month period preceding October 2021 [4], with the majority of overdoses involving opioids [4]. Stopping treatment and resuming opioid use after periods of abstinence is not uncommon for people with OUD [5]. This information is noteworthy because opioid overdose mortality risk is lower when patients are receiving treatment (e.g., methadone, buprenorphine) than after treatment. For instance, Sordo and colleagues conducted a meta-analysis of cohort studies and estimated overdose mortality rates per 1000 person-years to be 2.6 in methadone treatment and 12.7 after methadone treatment, and 1.4 in buprenorphine treatment and 4.6 after buprenorphine treatment [6]. Furthermore, as a result of reduced drug tolerance people with OUD are more likely to overdose after having periods of abstinence such as recently being released from jail or inpatient treatment [7–9]. While effective treatment is needed, historically, access to inpatient or outpatient specialty care substance use disorder (SUD) treatment in the United States was limited to those who could pay for treatment or had an insurance policy with treatment as a benefit [10]. However, legislative changes have changed the way SUD treatment is funded and have increased access to health insurance and SUD treatment for many Americans.

In 2008, Congress passed the Paul Wellstone and Pete Domenici Mental Health Parity and Addiction Equity Act (MHPAEA) [11]. In 2010, Congress passed the Patient Protection and Affordable Care Act (ACA) [11]. The combination of these two legislative changes fundamentally changed the way SUD treatment was funded. Before the enactment of the MHPAEA and ACA, not all insurance plans paid for SUD treatment and access to treatment was limited. Because the ACA allows young adults (19–25) to be on their parent’s insurance policy until they turn 26, this segment of the population has seen an increase in insurance coverage and subsequent access to SUD treatment [12] with uninsurance rates for young adults (19–25) decreasing to 14% in 2017 [13] from close to 30% in 2009 [10]. In general, the goals of the MHPAEA and ACA are to decrease the number of uninsured persons with OUD, decrease costs as a reason for not attending treatment, and increase the amount of out-of-pocket costs for SUD treatment paid for by insurance [14, 15]. These changes in healthcare policy have increased access to treatment but similarly to other healthcare sectors, abuses have been reported [16].

Fraud and abuses in the healthcare industry such as insurance fraud are not new. It has been estimated that economic losses associated with healthcare fraud, abuses, and waste are as high as $700 billion annually [17]. Furthermore, estimates have put healthcare fraud at anywhere between 3 and 10% of total expenditures for the industry [18, 19]. In the substance use treatment field, insurance fraud cases have increased, with more than $845 million in allegedly false and/or fraudulent claims to both private and federal health care programs connected to substance use treatment facilities or “sober homes” in 2020 [20]. Most notable is the rise in patient brokering fraud cases. Patient brokering falls under anti-kickback laws and can be defined as unlawful payment to an individual or business for the referral of a patient. Patient brokering can also include in-kind payments. In-kind payments may involve offering individuals goods or services, such as housing, transportation, or other material benefits, to encourage them to attend a particular treatment center or to remain in treatment. While some travel per California laws is allowed (e.g., ground transportation less than 125 miles) other types of travel are prohibited (e.g., one-way flight without a return ticket). Crimes related to violating anti-kickback laws have been prosecuted in fields such as home healthcare, pharmacy, physical therapy [21], and for-profit substance use treatment [20]. For example, the chief executive officer of Serenity Ranch Recovery in Florida was convicted of health care fraud and money laundering between 2016 and 2019 for fraudulently billing commercial insurance companies more than $36 million for substance use treatment services. Serenity Ranch offered free housing, cash, airline tickets, and copayment waivers to attract patients between the ages of 18 to 26.

Patient brokers are treatment center employees or work independently and get paid by treatment centers to exploit those seeking help for SUD. By making promises of luxurious treatment facilities and personalized care and buying plane tickets and facilitating the patient’s travel, treatment centers can significantly increase their revenue by admitting individuals with more lucrative insurance coverage [20]. From the perspective of out-of-state patients, the promise of a “free” or heavily subsidized trip for treatment can be tempting. However, they may not fully comprehend the implications of being part of a brokering scheme. In some cases, patients might be misled into accepting treatment options that are not suitable for their specific needs, simply because the treatment center has a financial incentive to admit them.

In September 2020, the Criminal Division of the Department of Justice announced the Sober Homes Initiative, which is the first coordinated enforcement in the Department of Justice focused on the substance use treatment industry. The project is led by the National Rapid Response strike forces in Los Angeles and Miami and was enacted to focus on schemes intended to exploit patients suffering from SUD [20]. While the media have documented these crimes, very little has been published in the academic literature that contextualize these experiences.

In 2018, Ashford et al. published a qualitative study that was designed to understand the barriers to good quality care in SUD treatment. They interviewed United States substance use treatment professionals and found several obstacles that made providing treatment difficult, including a lack of collaboration among co-workers, lack of recovery support services, and the rise in unethical practices in the field such as patient brokering [22]. While previous research has identified unethical practices as a barrier to providing services, no known study has studied patient brokering in depth from the perspective of patients and professionals. Therefore, we used qualitative methods with people with OUD and professionals in the SUD field to highlight personal experiences and perceptions regarding abuses in the for-profit treatment industry that could harm people with OUD and increase the risk of an opioid overdose.

## Methods

### Recruitment

Professionals in the SUD treatment field who work with patient’s with OUD and people with OUD were recruited by a combination of convenience, snowball, and theoretical sampling. Specifically, 20 professional participants were identified and recruited by professional contacts (n = 6), a referral from professional contacts or other participants (n = 10), attending professional meetings (n = 2), and Google searches (n = 2). To recruit participants with OUD, flyers with contact information were distributed to treatment centers, sobriety clubs, and other relevant places frequented by people with OUD. Snowball sampling methods, for both types of participants, consisted of asking participants to give study contact information to peers who might be interested in participating in the study. Once a potential participant contacted the primary author of the study, a few eligibility questions were asked. If eligible and participants were interested in moving forward with the study, arrangements were made to conduct the interview at the location of the participants’ choosing (e.g., coffee shop, work office). Additionally, theoretical sampling [23] was used to check the data and fill hunches that emerged from initial interviews and this led us to include an additional two professional participants working in public health for the city.

### Interview guide

An interview guide was developed for this study from fieldwork, academic literature, and news reports of unethical practices in the substance use sector (Supplementary material 1). The interview guide for participants with OUD and professionals differed, but the themes that emerged from the interviews tended to correspond. Furthermore, the interview guide was updated regularly to capture themes as they emerged from the data.

### Data Collection

One-on-one semi-structured interviews were conducted from November 2018 to May 2019 in Orange, San Diego, and Los Angeles counties. All interviews were conducted at a private location (e.g., library, coffee shop, park, office) of the participant’s choosing. Conducting interviews in the field is an acceptable practice and has been successfully deployed without jeopardizing the quality of the interviews [24, 25]. Interviews lasted about an hour and were audio-recorded. Saturation of themes occurred for both groups. The interviews were transcribed by two research team members (SC, BD). The primary author of this study conducted all interviews. Interviews were de-identified and participants were assigned a number. All transcripts used in the manuscript were reviewed for accuracy. All parts of the study were approved by the University of California, San Diego’s Institutional Review Board (approval no. 181,654). All patients provided written informed consent prior to enrollment in the study.

### Participants

A total of 40 participants (20 with OUD and 20 professionals) participated in the present study. Furthermore, 10 of the participants with OUD were Southern California residents before attending OUD treatment, and 10 participants with OUD were out-of-state residents before attending treatment in Southern California. Out-of-state residents came to Southern California to go to treatment and in most cases decided to live in Southern California. A combination of in-state and out-of-state residents were interviewed because a large percent of the SUD treatment population in Southern California came to treatment from out of the state.

Participants with OUD were eligible to participate in the current study if they had previously been in drug treatment at least once in Southern California after March of 2012. Further eligibility criteria for people with OUD were as follows: over the age of 18 at the time of the interview, opioids as the primary drug of use (e.g., heroin, OxyContin), self-reported misuse of opioids within the past 3 years, having health insurance at the time of treatment in Southern California, and English-speaking. Opioid misuse within the past 3 years was chosen to capture more recent use. Eligibility criteria for professionals were as follows: over the age of 18, work in the SUD treatment field, and English-speaking. Participants with OUD received $5 for participating in the current study, and no financial incentive was provided to professionals for participating in the study.

### Data Analysis

A grounded theory approach was used to identify emerging themes from the data [26]. For every transcript, coding was conducted in three stages and aided by NVivo 12 Plus software [27]. The first step of the analysis involved closely reading each transcript and conducting initial/open coding to identify important words, groups of words, and sentences that were later labeled into categories. Secondly, axial coding was conducted and involved conceptual linkage and descriptive linking of categories from the initial/open coding. The goal of axial coding was to compare and refine categories and discard categories that did not fit. Lastly, selective coding was conducted and involved identifying relationships between categories. The primary author of this study reviewed and analyzed all codes and then a second member of the research team (BD) reviewed the transcripts and codes to reach a consensus on the themes. Any disagreements were discussed before a consensus was reached.

## Results

### Participants

The mean age of the sample with OUD was 32.65 and ranged from 25 to 49 years old. Most of the sample with OUD identified as male (n = 14), with the remaining identifying as female (n = 5) or non-binary (n = 1). A majority of the sample identified as white only, with the remaining identifying as white and Hispanic (n = 2) or white and mixed-race (n = 1). A total of 12 participants reported that they were homeless, 5 reported that they were living in a sober living or treatment center, and the remaining 3 stated that they lived in an apartment. A total of 17 participants reported that they had had at least one opioid overdose in their lifetime, and 18 participants reported witnessing an opioid overdose at least once. Participants reported that they had overdosed a median of 3 times and witnessed a median of 4 overdoses. Participants had been to treatment multiple times, with a median of 6 treatment episodes reported. On average, participants reported using opioids for 13.68 years, ranging from 5 to 31 years. Most of the sample reported heroin (n = 19) as their primary opioid of use, and 1 participant reported that they primarily used prescription pills. A total of 8 participants stated that they were not currently using drugs at the time of the interview. Most of the sample reported that they had injected drugs at least once in their lifetime (n = 16).

Among the professionals who were interviewed, 14 identified as male and 6 identified as female. Professionals reported working in the field from 2 to 20 years and represented a wide range of positions. Among them, 8 participants held positions directly involved in SUD management, 4 of whom were medical doctors actively engaged in the treatment of SUD patients or occupied positions within relevant domains such as public health or correctional health. Additionally, 3 participants were SUD counselors, 2 were owners of SUD programs, 2 were staff members working in sober living facilities, and 1 participant was a nurse specializing in SUD care. A majority of the professionals identified as white (n = 16) with the remaining identifying as Asian (n = 2), Middle Eastern (n = 1), and black (n = 1).

### Main themes

Several themes and subthemes emerged when conducting interviews with participants with OUD and professionals that highlighted ethical concerns in the SUD treatment sector of Southern California. The themes that emerged in the study were discussed among most participants (See Table 1) and are as follows: (1) financial and material enticements, (2) encouraging drug use in the for-profit treatment sector, and (3) contributors to overdose risk (See Table 2). Throughout the interviews, some participants used the term “body broker” while other participants used the term referral when talking about patient brokers. These terms will be used interchangeably throughout the article.

**Table 1:** Thematically coded participant responses – all

Themes	Responses from OUD participants		Responses from professionals	
	N	(%)	N	(%)
Financial and material enticements	15	75%	12	60%
Encouraging drug use in the treatment sector	5	25%	7	35%
Overdose risk	7	35%	7	35%

**Table 2:** Themes and subthemes

Themes	Sub-themes
Financial and material enticements	• Plane tickets paid for by treatment centers or patient brokers • Patient brokers recruitment strategy. • Family members seeking help from patient brokers • Paid to go to substance use treatment by patient brokers or treatment centers • Monetary incentive to receive medication
Encouraging drug use in the treatment sector	• Using drugs before treatment is provided • Staff providing drugs to patients in treatment • Using drugs in motels prior to going to treatment • Incentivizing drug use • Jeopardize the recovery process
Contributors to overdose risk	• Patient brokers and overdosing • Revolving door • Using opioids after periods of abstinence

### Financial and material enticements

***Plane tickets paid for by treatment centers or patient brokers.*** The majority (7 out of 10) of participants who came to Southern California from out of state to attend treatment had their plane tickets paid for and/or booked by a treatment center or patient broker. One participant who had a flight paid for and booked one day after contacting a patient broker shared his feelings about the process.

*You got to be making a grip of money [lots of money] to fly a guy out here the next day and spend $730 on his ticket. Without knowing him or knowing if he’s going to stay or anything, you know.* (Male; 25, from NJ, Interview # 6)

***Patient brokers recruitment strategy.*** People are offered insurance coverage brought through the ACA healthcare marketplace so they can enter treatment facilities, as the facility and patient broker stands to make a significant profit from their stay, despite the initial expenses. Patient brokers are often paid directly by the treatment center for each patient they can get to attend and stay in treatment and they often try to get others to recruit potential clients to maximize their profits.

*I know people saying, we’ll buy your plane ticket, do you have any friends that are in other states that want treatment? We’ll help pay for all this, cause what they’ll do is they’ll just go buy you an insurance policy. They’ll put $500 down; they’ll pay for your insurance. Even if it’s three months, they’ll pay 1,500 bucks, buy you a plane ticket. Let’s say that’s 500 bucks. It cost them two grand. But when they’re going to make $30,000 profit off you staying there for that time, so that person’s going to get 10 grand or whatever. So he’s gonna put his two grand up, no problem.* (Male; 31, from KY, Interview # 20)

Professionals’ stories of patient brokering in substance use treatment often parallel the stories discussed by participants with OUD.

*I have a huge problem with the treatment industry as of late because of all the insurance fraud, and the body brokering. They treat these kids like mules. I’ve watched staff, they aren’t staff members anymore at [Sober Place] for that reason, but I have watched a staff member body broker a client in front of my eyes.* (Male; house manager of sober living, Interview # 32)

***Family Members seeking help from patient brokers.*** While patient brokers often target individuals seeking treatment, they also target family members who want help for their loved ones. One participant shared that her family member followed a link on social media because they wanted to get the participant into SUD treatment, saw a post about a page that included numerous positive posts about it from others and how effective it was for their family members, and made contact with a patient broker who arranged to get the participant health insurance.

*She did everything [patient broker] and my aunt paid her and then one day I just opened the mail in September, and I had an insurance card there. (Female; from TN, Interview # 9)*

***Paid to go to substance use treatment by patient brokers or treatment centers***. Many participants with OUD reported that they received money or were offered money from patient brokers or treatment centers to go to treatment. One participant stated that they went into inpatient substance use treatment the last time because *“I needed money”* and reported getting $4,000 for attending treatment.

*So yeah, I called one of them, and he said I will give you $4,000 to go in for 20 days. (Female; 25, from NY, interview # 14)*

Financial enticements were especially important given the financial and living situations of some participants. Many participants felt that being offered money to attend treatment was unethical, but some participants felt that their choice was limited because of their situation. Participants often stated that they were unhoused, without money, and struggling with their SUD, and being offered money to attend treatment was an easy choice. For example, one participant said that they took the money when offered to get into treatment by a patient broker because they needed the money and a place to live.

*Yeah. I mean, I was broke doing drugs, and someone offers you a couple of thousand dollars and a place to live, it’s like, of course. So, I was in a bad spot and when I was in these places, it was, it seemed like 90% of the people were getting paid.* (Male; 27, interview # 11)

The same participant described a recent situation with their roommate at the treatment facility who was newly sober, unfamiliar with the body brokering process, and enticed by the financial and housing aspects of brokering, demonstrating the potential vulnerability of individuals newly sober who might be targets of body brokers and unable to say no to the temptation given their financial situation.

*He wanted to do it [receive money to go to a different treatment center]. And I talked him out of it. I was like ‘cause he can’t even afford cigarettes right now. He has no money, and someone comes and offers him two thousand dollars, and he has no money, and he’s like, “Oh, I’ll still have a place to stay and food to eat. What’s the difference between being at this treatment rather than this treatment?” The only thing is you have to go get high for a couple of days. And he’s new in his sobriety and he’s like, “Someone offers me money and drugs and I’m just supposed to say no?”. (Male; 27, interview # 11)*

Some of the participants also reported receiving more money from patient brokers or treatment centers the longer they stayed in treatment. For example, one participant, who has been paid to go to substance use treatment numerous times, described how much they would get paid when he went to treatment.

*Two grand here, 3 grand there, 15 hundred here. It depended on how long I stayed, and the program or the contract. You know it all depends, not only that but the program would put me in a motel for a couple of days you know, food, money.* (Male; 25, from JN, interview #6)

***Monetary incentive to receive medication.*** One participant reported being compensated several times to have extended-release naltrexone implanted under his skin. He believed the medication was ineffective because it was easy to surgically remove after the procedure.

*You’d go in, they’d put the pellets in you, and then you’d walk out. It would take all of 15 min. So, I walked in, I got the pellets put in me, I had a buddy take them out, and I would go back in and get them put back in. I’d get paid by 3 different people.* (Male 25, interview # 18)

Reports of receiving financial enticement to get naltrexone implants was a theme shared by professional participants as well. Here a participant in the medical field shares her thoughts regarding naltrexone implants.

*Then you’ve got the other thing that comes up, is you have the implants, the naltrexone implants. Total body brokering type of thing. Patients getting paid to do it. Pellets not being good, pellets probably not being even a pellet. (Female: Nurse, interview # 23)*

### Encouraging drug use in the treatment sector

***Using drugs before treatment is provided.*** Some of the participants discussed that they were required to use drugs before they could get into treatment. Participants explained that insurance companies require certain conditions to be met so that the patient meets medical necessity and treatment will be paid for by the insurance company. Recent drug or alcohol use and providing a positive urinalysis often would meet insurance companies’ requirements for a new treatment episode so that treatment would be reimbursed. This happened most often when participants were seeking detoxification or inpatient treatment. A participant who had paid others to attend treatment and who had been paid to attend treatment describes the process.

*It does not necessarily mean you have to get loaded when you go into treatment, but there’s only certain substances you put in your body that would qualify you for medical detoxification. So, if you don’t at least piss dirty for one of those things, then no, they won’t take you. (Male; 27, from Connecticut interview # 19)*

***Staff providing drugs to patients in treatment.*** Counselors discussed how they’ve heard about or witnessed staff who participate in providing drugs to their clients who are in treatment so that the patient would test positive for substances and can stay in treatment longer or get back into treatment after being discharged.

*There’s been reports that staff has participated in providing drugs to clients. Call me crazy -- that sounds like a problem. You have the issues with the body brokers and that kind of stuff. Pulling people out of treatment, getting them high and then getting back into treatments so that they can make more money.* (Male; counselor, interview # 22)

Another professional participant reports a similar unethical conduct and stated instances of counselors giving patients drugs.

People are dying from not getting real quality treatment. People are literally dying from being taken out and getting high. I’ve heard of counselors having drug doors [drugs for patients]. (Male; Director, interview # 21)

***Using drugs in motels prior to going to treatment***. One participant who was administratively discharged from an inpatient treatment center for verbal misconduct discussed how he used drugs in a motel before being allowed to go to a different treatment center. The participant reported that his broker arranged a motel stay and suggested he use legal or illegal drugs prior to admission so that he could be eligible for inpatient treatment.

*Yeah, then came to the hotel. We had to start over again for the program that I was going to, so we had to fail a drug test somehow. So, we just drank and smoked some weed and then I went to [Big Pine].* (Male; 36, from Boston, interview # 1)

Professional participants who were aware of the practice were particularly distraught by the unethical conduct that was happening in the industry. Professional participants believed that people were being bought and sold for profit.

I know a lot of people who take clients and like put them in hotels and get them all fuc*** up on heroin and then sell them to treatment centers. (Male; house manager for sober living, interview # 32)

***Incentivizing drug use.*** Some professional participants also believed that the practice of body brokering was incentivizing the return to drug use.

*They’ll influence the kids [patient brokers] at that program to leave the program, relapse and then they’ll pay them money to come over to this other program. And what happens is that these kids, it’s called Rehab Surfing. They have developed this very strong habit, if not addiction to relapse because it’s being incentivized with money. And now they have a roof over their head and it’s like a vacation. But you have to relapse continually to keep a roof over your head.* (Male; treatment center owner, interview #24)

***Jeopardizing the recovery process.*** A few participants stated that paying people to attend treatment jeopardizes the recovery process and adds another layer of temptation in addition to the temptation to use opioids. One participant who was not using drugs at the time of the interview describes the added temptation that she experienced knowing she could get paid to resume drug use and then go into treatment.

It terrifies me because I know that with my disease like at any point in time it’s just waiting for me to have a thought of, I could get paid to do what I love to do which is using drugs, and that terrifies me because I know I don’t want to but if that’s an option, it’s always gonna be an option. You know, I mean. It fuc*in’ terrifies me. So I know I’ve got to do what I do today to make sure that that stays where it stays. But it does scare me that that’s an option today. And it’s scary. It’s going to kill a lot of people. (Female; 36, interview # 5)

I mean, I’ve been offered to go get paid like a thousand bucks to go get the shot and that was when, you know, I just got out of residential, I wasn’t working, and I didn’t have a job. You know, no money. So, that was like really tempting. But I didn’t fuc*in do it because I didn’t want. I just felt like I was in the mindset that everything I do is a potential risk or potential threat so my life was that serious. (Male; 25 from NJ interview # 12)

***Contributors to Overdose Risk***.

**Patient Brokers and Overdosing.** A significant number of participants pointed out the linkage between patient brokering and opioid overdose deaths in Southern California. One participant believes the unethical treatment environment as a result of patient brokering exacerbates this risk.

*It still sits sour with me, just on the principle of it… A lot of people, kids, kids have died cause of that. I mean I just figure they come out of treatment to get paid… they get all this money and they OD and die.“ (Male, 39, from Ohio, interview # 15)*.

Some professionals linked the detrimental treatment environment to opioid overdose deaths, emphasizing the role of patient brokers in supplying drugs.

*What I do know is that these very high-powered drugs are being provided to people that like to use them and death is happening as a result. I’ve been talking to - if you really want to have some interesting conversations - I have a little group of moms that I’ve been working with and all lost kids and most of them believe that they lost kids as a result of shoddy treatment. These overdose deaths, right? Most of them. So yeah, so I think that there is, I believe that in, in some ways addiction treatment professionals are complicit in the death of young people that didn’t need to die.* (Male; counselor, interview #22)

Most of the participants who discussed overdose risk in the context of unethical treatment believed patient brokering was the leading cause. A few of the participants discussed instances where patient brokers would infiltrate treatment centers attempting to recruit more people to go to a different treatment program for profit. Because the broker takes the recruit to a motel after leaving treatment to use drugs, he becomes vulnerable to an overdose.

*They take them out of treatment centers, and they put them in motels and get them high. So, I mean, I know personally that there have been a few clients that have overdosed in rooms with body brokers.* (Male; house manager for sober living interview # 32)

***Revolving door.*** A few professional participants felt that the revolving nature of substance use treatment and the increased access to treatment as a result of insurance coverage created an environment where clients no longer prioritized recovery and each time they go back to using drugs they risk overdosing and dying.

*Well, that’s the problem, because essentially, since these kids are now in a revolving door, eventually they’re going to take a hotshot [lethal dose] and die. We have had more deaths in the last two or three years than I’ve seen in 15 previous years. (Male; Director of a treatment program, interview # 33)*

***Using opioids after periods of abstinence***. Several participants highlighted the danger of using opioids after periods of abstinence, especially when given drugs before or between treatment episodes. Some participants believed being given drugs before attending treatment or between treatment episodes contributed to opioid overdoses as drugs were provided after periods of abstinence.

*Yeah, I mean, I almost died the first time I ever shot heroin, like, you know. And if, especially if you’ve been clean for a while and somebody offers you like right now like I have a hundred and fifteen days, and if somebody offered heroin, you know for me like there’s a great chance that I would overdose and die.* (Female; 36, interview # 5).

## Discussion

Our study explored abuses in the for-profit treatment industry as experienced by those who lived it. Interviews with people with OUD who attended SUD treatment and professionals who work in SUD treatment yielded several themes that demonstrate unethical abuses in the SUD treatment sector are harmful and could potentially impact a person’s risk for an overdose. According to interviews, patient brokering has caused some people to seek treatment for reasons other than getting help for their OUD. For instance, participants with OUD reported seeking treatment for survival rather than to address their drug use. Many participants with OUD perceived that the harmful treatment environment has made getting real help for their drug use harder and that by adding financial incentives to attend treatment, the integrity of the treatment process is compromised. Other participants, who were tempted by offers of money to attend treatment, refused, stating that they wanted to take their recovery seriously and that their life was on the line. A few participants who did not go to treatment for money felt that the temptation caused by patient brokers made it harder to stay away from drug use because the temptation to use for profit was an option. Others who received legitimate treatment services through patient brokering schemes benefited from access to insurance and treatment but the unethical behavior likely minimized the positive impact of treatment services received.

Our results show patient brokering and unethical abuses in SUD treatment have been harmful and a barrier to care and are in line with previous research with professionals [22]. For instance, many of the participants believe that patient brokering, financial enticements, and encouraging drug use before attending treatment are responsible for fatal and non-fatal opioid overdoses in the treatment-seeking community. While some participants believed the unethical abuses in the treatment industry have directly caused opioid overdoses, others believe that the harmful treatment environment has created conditions where an opioid overdose is more likely. Many professional participants believe real treatment is not being provided by the unethical programs and, as a result, some people have died. Many of the participants with OUD perceived that the risk of overdose was greater after attending unethical treatment, in part because of the money that was given after attending treatment. Specifically, some participants believed that the money given elicited a desire to use opioids and provided a financial opportunity to purchase large amounts of drugs. Past research has shown having too much money can elicit a desire to use [28], and opioid overdoses are more likely after periods of abstinence [7, 9], making these unethical practices a deadly combination for those with OUD. Furthermore, those with OUD often return to opioid use soon after treatment, [5] further complicating the issue and making it even harder for those seeking treatment.

### Strengths and limitations

We have contextualized how unethical abuses in the for-profit SUD treatment sector may have negatively impacted people seeking treatment from the perspective of patients and professionals. We recruited a diverse treatment sample of participants who shared their experiences and insight on an important topic. Only English speakers were recruited for the present study, so generalization to other groups is not possible. A majority of the sample was largely homogeneous, and findings can’t be generalizable to other sociodemographic groups. It is therefore unknown the degree to which the participants were representative of all people who attended SUD treatment. Furthermore, the coding of narratives was primarily conducted by one person and therefore, unintentional biases may exist. However, these codes were reviewed by another member of the research team (BD) to reach a consensus on the themes. Finally, no explicit determination can be made of the cause and effect of opioid overdoses as a result of unethical treatment, given the nature of the study.

### Conclusions and recommendations

The exploration of the for-profit treatment industry reveals profound concerns regarding the unethical practices that not only hinder effective recovery but also heighten the risk of overdoses among individuals with OUD. Our study underscores the dangerous ramifications of patient brokering, where individuals are driven to seek treatment not for genuine recovery but due to external pressures or financial enticements. The revelation that many of our participants feel that financial incentives compromise the authenticity of the treatment process further emphasizes the need for radical changes in the SUD treatment sector. The collective narratives from both treatment seekers and professionals in the SUD treatment sphere point towards an urgent need to address these unethical approaches, as they not only hinder genuine recovery attempts but can lead to fatal consequences. As we move forward, it is imperative to prioritize the integrity and efficacy of treatment approaches, ensuring they cater to the genuine well-being and recovery of individuals with OUD, rather than being driven by profit-driven motives.

To effectively address the highlighted issues, there must be a concentrated effort on bolstering regulatory oversight and promoting the reporting of unethical practices. Bringing greater awareness to these issues and normalizing the reporting of unethical practices through reporting systems would likely curb patient brokering and foster a treatment environment that focuses on patient care. Additionally, an emphasis on training and education to ensure providers understand their ethical responsibility and are using evidence-based practices should be a priority. Evidence-based treatments, particularly medications for OUD, are underutilized in substance use treatment facilities, with a majority of them not offering such options [29]. Yet, it’s worth noting that in states where there’s an expansion of Medicaid and broader access to medication treatments, facilities are more inclined to provide medications for OUD [29]. This underscores the potential benefits of broadening health insurance access and providing quality care under diligent oversight, propelling more facilities to embrace best practices. Finally, our findings underscore the critical role of housing access in the context of SUD treatment. A substantial number of participants reported being homeless or living in treatment centers, highlighting the intersection of housing instability with substance use challenges. Furthermore, the allure of financial incentives, often presented as a pathway to secure housing, illustrates how vulnerable populations can be drawn into potentially unethical treatment practices. Ensuring stable housing access can thus not only provide a foundational element of security for those with SUD but also mitigate the allure of potentially exploitative practices in the treatment industry. These recommendations are crucial steps towards creating a safer, more ethical, and effective SUD treatment environment that genuinely prioritizes the well-being and recovery of individuals with SUD.

### Electronic supplementary material

Below is the link to the electronic supplementary material.

Supplementary Material 1

## Data Availability

The data is not publicly available to protect the confidentiality of the participants. Data is available upon reasonable request to Sarah E. Clingan.

## Abbreviations

ACA: Affordable Care Act
CDC: Centers for Disease Control and Prevention
MHPAEA: Paul Wellstone and Pete Domenici Mental Health Parity and Addiction Equity Act
OUD: Opioid use disorder
SUD: Substance use disorder
