# Supplementary material for: Patient brokering in for-profit substance use disorder treatment: a qualitative study with people with opioid use disorder and professionals in the field
Source: BMC Health Serv Res. 2023 Nov 6;23:1214. doi: 10.1186/s12913-023-10217-z (PMC10629128; doi:10.1186/s12913-023-10217-z)
Supplement: Supplementary file 1 — Supplementary Material 1 [file 12913_2023_10217_MOESM1_ESM.docx]

Interview Guide

**Participant Type:** People with opioid use disorder

1. What is your age?

2. What gender do you identify with?

3. What is your race/ethnicity?

4. What is your living situation like?

5. How long have you been using drugs?

6. Tell me about the last time you went to inpatient drug treatment.

7. Have you attended other types of drug treatment (e.g., outpatient or sober living)?

8. Tell me why you went into drug treatment.

9. How did you pay for drug treatment?

10. Did you ever experience body brokering (e.g., someone else getting paid for you to attend drug treatment) or other types of unethical behavior while you were in drug treatment?

11. Did you ever get paid to attend drug treatment or detox?

12. Tell me about the positive experiences you had while you were in treatment.

13. Tell me about the negative experiences you had while you were in treatment.

14. Tell me about your experiences with opioid overdoses. Have you witnessed an overdose?

15. Have you had an opioid overdose?

16. Have you ever received naloxone (trade name Narcan) the drug given to someone when they are overdosing? Tell me about your experiences.

17. Have you ever received education or training about had to respond to an opioid overdose in drug treatment?

18. Have you ever received Narcan after leaving drug treatment?

19. Have you or others you know encountered any stigma related to drug treatment?

20. Do you think different types of drug treatment are stigmatized differently, for example, NA vs methadone or MAT?

21. If you wanted to go back into treatment what might prevent you?

22. What can we do about some of the problems you discussed? What are the solutions?

**Participant Type:** Professionals

1. What are the effects of MHPAEA and ACA on opioid overdoses?

2. How effective are MHPAEA and ACA on intermediate effects (i.e., more people going to treatment)?

3. Is the intervention logic of this policy plausible?

4. How does the implementation context influence this policy’s effectiveness?

5. How much time is needed before effects can be observed? Do the effects persist over time? When do you notice changes in the system post-ACA?

6. Does the policy under study produce unintended effects, whether positive or negative (e.g., have treatment centers been unethical as a result of more funds for treatment)?

7. How can the negative unintended effects be mitigated?

8. What are the effects (intended or unintended) of the policy under study on different groups (e.g. access to healthcare might be different for different racial groups)?

9. Does this policy create, reinforce, or correct social inequalities in health?

10. Can you tell me about the use of Narcan in the industry?

11. Can you tell me about MAT in the industry?

12. Stigma with MAT?

13. Can you tell me about the education level in the field?

14. What type of evidence-based approaches are used in the field?

15 What can we do to address the problems you discussed today? What are the solutions?
